# Development and Long–Term Operation of a Three-Dimensional Displacement Monitoring System for Nuclear Power Plant Piping

**DOI:** 10.3390/s26030895

**Published:** 2026-01-29

**Authors:** Damjan Lapuh, Peter Virtič, Andrej Štrukelj

**Affiliations:** 1 Nuclear Power Plant Krško, Vrbina 12, SI-8270 Krško, Slovenia; damjan.lapuh@nek.si; 2 Faculty of Energy Technology, University of Maribor, Hočevarjev Trg 1, SI-8270 Krško, Slovenia; peter.virtic@um.si; 3 Faculty of Civil Engineering, Transportation Engineering and Architecture, University of Maribor, Smetanova 17, SI-2000 Maribor, Slovenia

**Keywords:** nuclear power plant, piping systems, displacement monitoring, inductive transducers, data acquisition, radiation environment, structural integrity, experimental diagnostics

## Abstract

Ensuring the structural integrity of high-energy piping systems is a critical requirement for the safe operation of nuclear power plants. This paper presents the design, implementation, and three-year operational validation of a three-dimensional displacement monitoring system installed on the steam generator blowdown pipeline of the Krško Nuclear Power Plant. The system was developed to verify that the plant’s operating procedures will not induce excessive dynamic displacements during operation. The measurement system configuration utilizes three non-collinear inductive displacement transducers from Hottinger Baldwin Messtechnik (HBM WA/500 mm-L), mounted via miniature universal joints to a reference plate and to a defined observation point on the pipeline. This arrangement enables the real-time monitoring of X, Y, and Z displacements within a spherical measurement volume of approximately 0.5 m. Data are continuously acquired via an HBM QuantumX MX840B amplifier and processed using CATMAN Easy-AP software through a fiber-optic communication link between the containment and control areas. The system has operated continuously for more than three years under elevated temperature and radiation conditions, confirming its reliability and robustness. The correlation of the measured displacements with process parameters such as the flow rate, pressure, and temperature provides valuable insight into transient events and contributes to predictive maintenance strategies. The presented methodology demonstrates a practical and radiation-tolerant approach for the continuous structural monitoring of nuclear plant piping systems.

## 1. Introduction

This article addresses the behavior of high-energy piping systems and the mechanisms that govern pipe movement and induced stresses under operation conditions. After presenting the special developed sensor setup and the mathematical background, the actual system setup is presented. The article later presents some measured data and ends with some conclusions.

At the Krško Nuclear Power Plant (NPP Krško), a specific technical challenge has persisted for several years: to prove that regular operator actions and unpredicted transients in the steam generator blowdown (BD) system will not generate excessive dynamic forces that cause excessive pipe displacements. These operating procedures and transient conditions are defined in the plant documentation [1], and the monitoring system was developed to support their verification by ensuring that such operating scenarios do not induce excessive dynamic displacements of the piping system. As in all high-energy piping systems, the BD system is supported by a combination of fixed and dynamic hangers. These supports accommodate thermal expansion while simultaneously mitigating transient dynamic loads [2]. Ensuring the structural integrity of such piping demands considerable engineering effort to achieve adequate support that allows controlled movement, maintains overall stability, and prevents damage under both normal and off-normal operating conditions.

**Figure 1:**
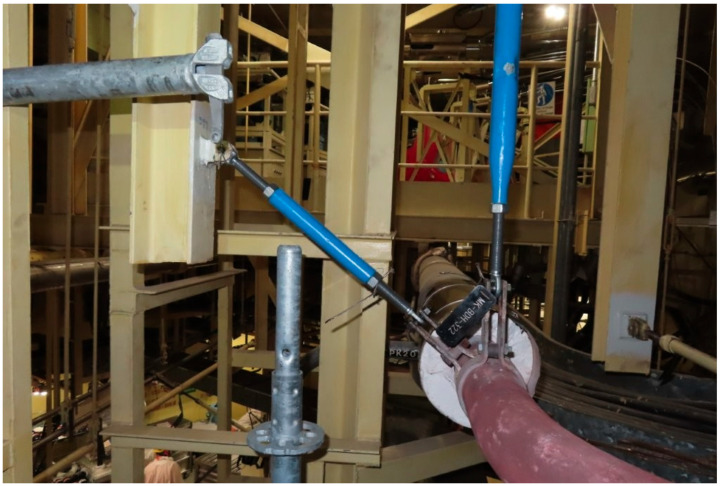
Typical sway struts (blue) supporting the BD system piping at the NPP Krško.

**Figure 2:**
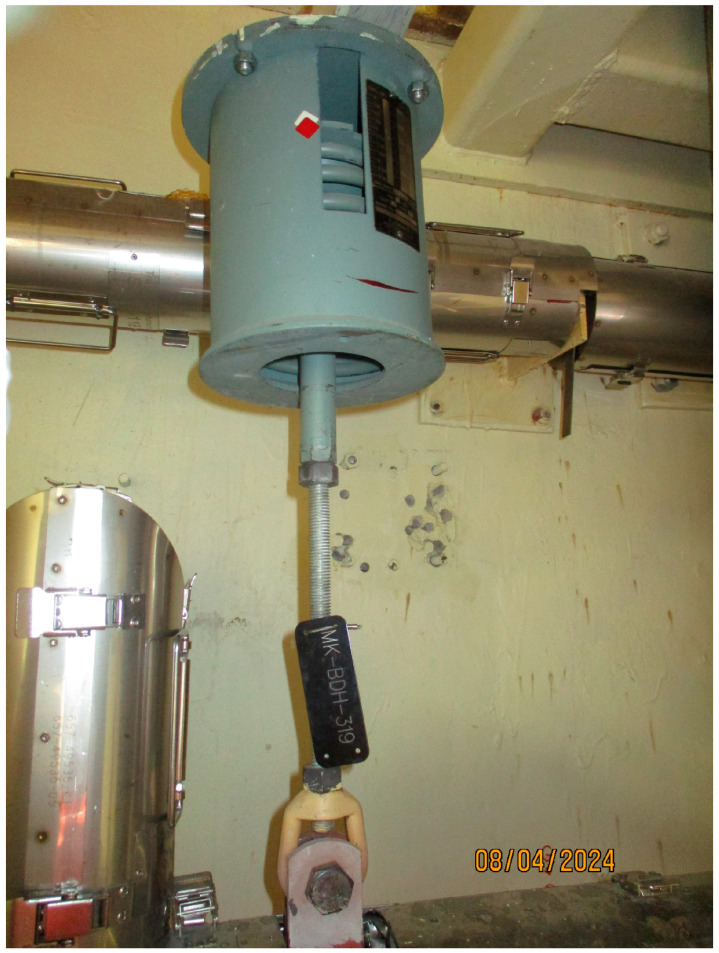
Variable spring, hanging from the top console and supporting the pipe on the bottom.

Support components are routinely subjected to regular tests and visual inspection for signs of degradation or abnormal behavior [3]. Figure 1 and Figure 2 show typical pipe support components—sway struts and variable springs. Because the BD piping is located within the containment building, such inspections can be performed only during scheduled refueling outages, which occur every 18 months. Entry into containment during power operation is strongly discouraged due to the elevated temperature, humidity, and most importantly, radiation levels.

Pipe displacement in power plants can arise from several interacting factors, each contributing to the overall dynamic behavior of the piping system:

- Thermal expansion and contraction: Temperature variations within the piping system can cause the material to expand or contract. As the temperature of the conveyed fluid or the surrounding environment increases, the pipe expands; conversely, it contracts when the temperature decreases. These cyclical thermal deformations can induce movement and additional stress in the piping network, particularly at locations with constrained boundary conditions or insufficient flexibility [4].
- Vibrations and dynamic loads: Operational equipment such as pumps, valves, turbines, and compressors generate vibrations and transient dynamic loads. These loads can be transmitted through the piping system, leading to displacements and localized stress concentrations. Under adverse operating conditions, such excitation may lead to resonant vibrations, which can amplify the mechanical response and accelerate fatigue or wear of the support components [5].
- Pressure fluctuations: Rapid pressure changes can also induce significant pipe movements. Pressure surges, water hammer events, or abrupt variations in the flow rate produce hydraulic forces that can push or pull the piping beyond its expected limits if the system is not adequately supported or damped [6].
- Seismic events: For power plants located in seismically active regions, ground motion due to earthquakes is a major source of dynamic excitation. Seismic loading can induce substantial pipe displacements and impose additional stress on supports and connecting elements. Proper seismic qualifications of supports and structural anchorage is therefore essential to mitigate these effects and to ensure the system integrity during seismic events [7].

To identify under which operating conditions these issues occur and to quantify the actual pipe displacements during plant operation, a dedicated monitoring system for displacement measurement was designed and implemented.

The principal objectives in developing the measurement system were as follows:

- To enable the precise real-time measurement of displacements in all three spatial directions (*X*, *Y*, *Z*).
- To ensure automatic data acquisition and archiving with subsequent analytical capability.
- To provide continuous access to measurement data without requiring physical entry into the containment building.

The aims of this study were to develop a robust and reliable methodology for measurement, data acquisition, and processing suitable for industrial application under elevated temperature, humidity, and radiation conditions. To the best of the authors’ knowledge, this is the first in situ implementation of such a measurement approach within a nuclear power plant environment and is the first of its kind applied at the NPP Krško.

**Figure 3:**
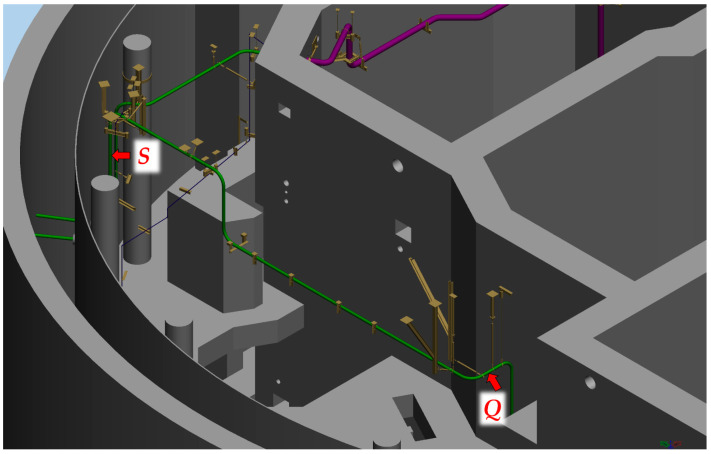
Location of the measuring points S and Q inside the reactor building.

A comprehensive review of the available technical literature and industrial applications revealed very few comparable systems that had been implemented on a high-temperature steam pipeline in a gas-fired power plants [8,9]. The referenced systems employed a robotic-arm-type three-dimensional displacement sensor comprising two rigid arms connected to a fixed base and equipped with two angular sensors and one rotational sensor. Pipe displacement was derived from the fixed known arm lengths and the measured variations in the joint angles. A second system followed a similar principle, consisting of rotational and angle sensor; however, instead of two rigid arms with known fixed lengths and an encoder, it employed a single variable-length arm. This arm integrated a linear displacement sensor, enabling pipe displacement to be determined from the measured rotation, known angle, and measured length. In both cases, angular measurements were obtained using digital encoders, while linear displacements were measured using an LVDT sensor. The system developed in this study utilizes linear length measuring sensors, mounted in a mutually non-collinear configuration. One end of each sensor is attached to a common point connected to the monitored pipe, while the other end is fixed to a known point on a mounting plate. Among various available linear displacement sensors, the LVDT type was chosen because of its proven robustness, reliability, high resolution, and ability to retain position information in the event of a power failure [10]. All LVDT sensors are connected to a data acquisition (DAQ) unit that is linked to a PC computer. Due to the long distance and limited cabling options from the reactor building, optical fiber communication was employed. A PC with running legacy software collects, calculates, displays, and archives the data. A measuring system monitors two points, S and Q (Figure 3).

To the best of the authors’ knowledge, although studies have been conducted on thermal power plants [8,9], displacement measurements of piping systems in nuclear power plants have not yet been widely adopted. More recent power plants primarily focus on measuring vibrations and accelerations [11].

## 2. Materials and Methods

The spatial position of a point is fully defined by three translational degrees of freedom and can therefore be uniquely described by three coordinates in an arbitrary coordinate system. In the proposed measurement concept, this requirement is satisfied by employing three non-collinear displacement transducers, each providing an independent measurement of the change in distance along a distinct spatial direction. Together, these three measurements define a local, non-orthogonal coordinate system. As long as the sensor axes are not collinear, the corresponding measurement model has full rank, and the transformation from the sensor-based coordinate system to the Cartesian X–Y–Z coordinate system is unique. Consequently, the three-dimensional displacement components of the observed point can be fully observed and uniquely reconstructed.

The use of miniature universal (cardan) joints at both ends of each transducer ensures that only the axial distance between the attachment points is measured, while allowing free rotation of the transducers. This configuration prevents the introduction of parasitic bending or additional kinematic constraints that could otherwise reduce the observability of the system.

Due to their reliability, linearity, and robustness [10], linear variable differential transformers (LVDTs) were selected as the basic elements of the measurement chain. The inductive displacement transducers used in the system are WA/500 mm-L sensors produced by Hottinger Baldwin Meßtechnik (HBM), Darmstadt, Germany. Transducers of the WA-L series with a loose plunger design provide high measurement precision, excellent linearity, and stable temperature behavior [10]. Their compact construction—achieved through an integrated differential inductor full-bridge circuit—allows direct connection to the measuring amplifier and ensures robust performance in demanding industrial environments, including insensitivity to dirt and mechanical wear.

Inductive displacement transducers are inherently designed to measure displacement along a single axis. However, the movement of the observed point on the pipeline follows a spatial trajectory. While it would theoretically be possible to determine the position of the observed point using a single displacement transducer combined with simultaneous angle measurements, such an approach would require continuous real-time measurement of two additional angular degrees of freedom. This is impractical for long-term monitoring in a nuclear power plant environment. For this reason, the spatial position of the observed point is determined by simultaneously measuring three mutually non-collinear displacements using three inductive displacement transducers.

For each observation point, the system consists of three LVDTs mounted on one side to a rigid steel plate representing the reference plane. The plate is installed vertically, parallel to the straight section of the monitored pipeline. The attachment points of the transducers on the reference plate ($T_{1}$, $T_{2}$, and $T_{3}$) are arranged at the vertices of an equilateral triangle. On the opposite side, the transducers are connected to the pipeline at the observation point P using a specially designed clamp, which allows the sensor axes in the initial position to intersect as closely as possible at the monitored point. Universal joints are used on both sides of the transducers to accommodate relative rotations during pipe movement.

**Figure 4:**
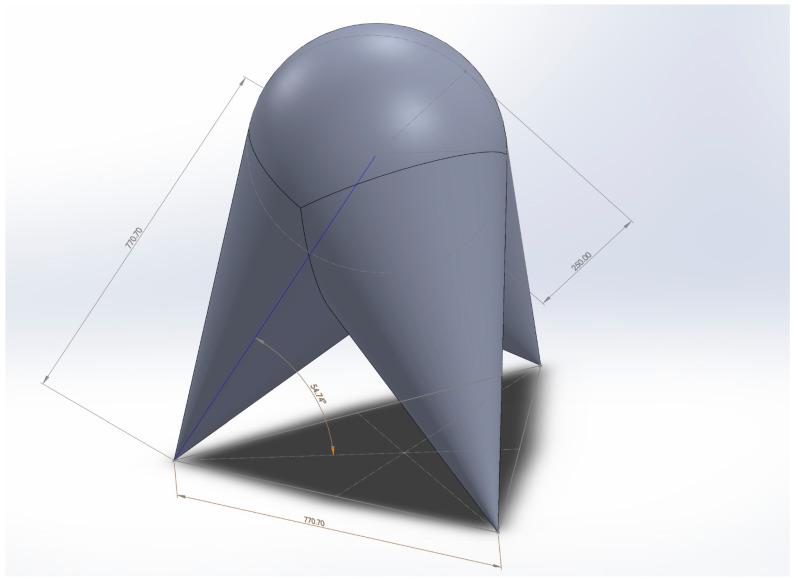
Spatial representation of the area within which the inductive displacement transducers and the observed point move.

To ensure unobstructed operation, the spatial domain of possible transducer and observation-point positions was defined in advance in the form of a sphere and three cones, which together describe the volume within which no obstacles may be present (Figure 4). Based on this geometric analysis, the required measuring ranges of the individual system components were determined.

**Figure 5:**
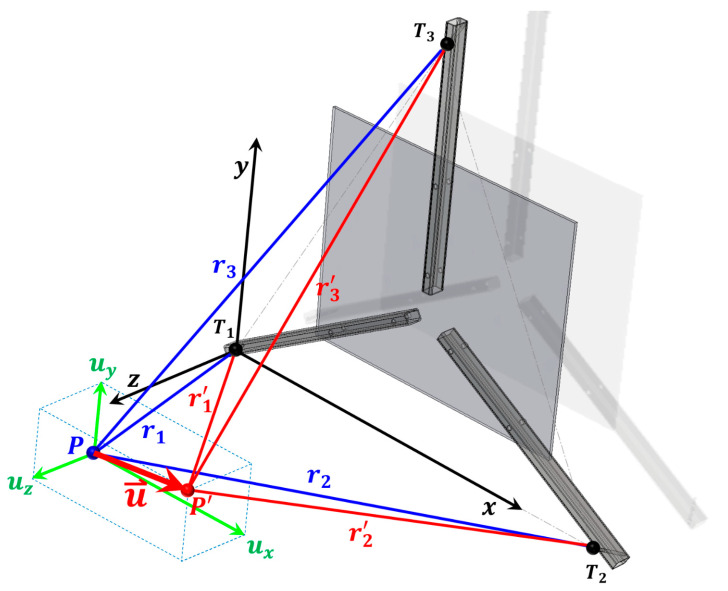
Geometric representation of the measuring system during the displacement of point P to $P^{\prime }.$ The initial position of point P and the corresponding distances to the fixed points $T_{1}$, $T_{2}$, and $T_{3}$ are shown in red. The displaced configuration after the movement in the direction of the displacement vector $\vec{u}$ is shown in blue, while the displacement components $u_{x}$, $u_{y}$, and $u_{z}$ are highlighted in green.

**Figure 6:**
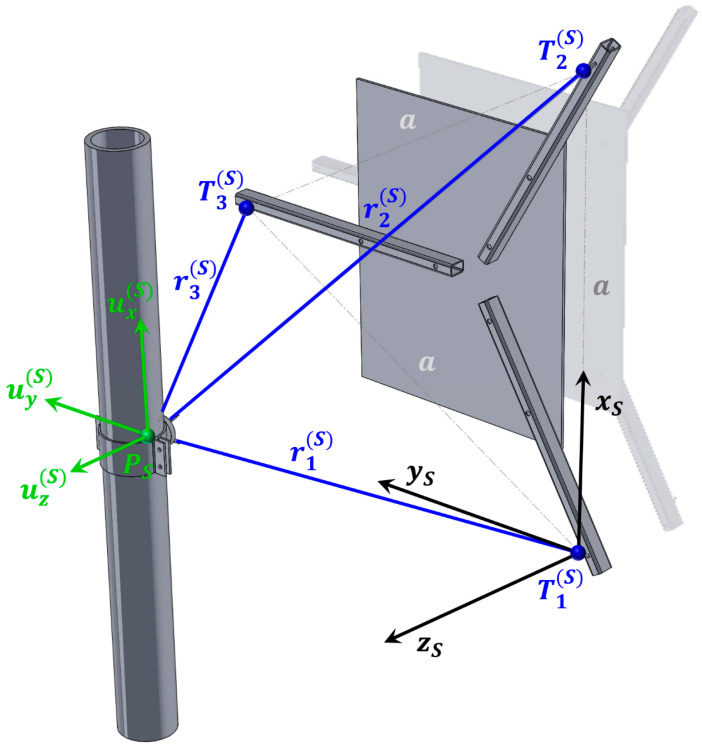
View of the measurement system at location S.

**Figure 7:**
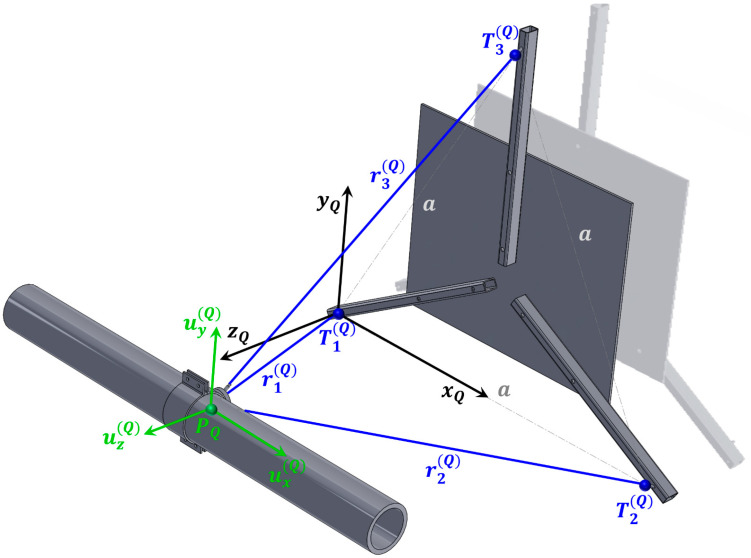
View of the measurement system at location Q.

When the observation point moves from position P to P’, the three transducers measure three independent, non-collinear changes in distance. From these measurements, all three displacement components are determined in the defined coordinate system (Figure 5, Figure 6 and Figure 7). The coordinate system is defined such that the x-axis lies along the line connecting points $T_{1}$ and $T_{2}$, the y-axis is perpendicular to the x-axis within the plane defined by points $T_{1}$, $T_{2}$, and $T_{3}$, and the Z-axis is oriented perpendicular to the X–Y plane, forming a right-handed Cartesian coordinate system.

Because the measurements are performed in a highly radioactive environment that is inaccessible during reactor operation, the measurement system must be exceptionally robust and capable of reliable operation over the entire range of theoretically possible displacements. 

The displacement components of point *P*, denoted as *u_x_*, *u_y_*, and *u_z_*, are aligned parallel to the respective coordinate axes (see Figure 5).

Since the origin of the coordinate system is placed at point $T_{1}$, based on Figure 5, we can write Equations (1)–(3), which represent a system of equations from which the expressions for the coordinates of the point $P(x_{P},y_{P},\;z_{P})$ can be determined:

(1) $$r_{1}^{2}=x_{p}^{2}+y_{p}^{2}+z_{p}^{2},$$

(2) $$r_{2}^{2}={\left(x_{p}-x_{2}\right)}^{2}+y_{p}^{2}+z_{p}^{2},$$

(3) $$r_{3}^{2}={\left(x_{p}-x_{3}\right)}^{2}+{\left(y_{p}-y_{3}\right)}^{2}+z_{p}^{2}.$$

From the difference between Equations (1) and (2), we obtain

(4) $$x_{p}=\frac{r_{1}^{2}-r_{2}^{2}+x_{2}^{2}}{2x_{2}}.$$

From the difference between Equations (2) and (3), we obtain

(5) $$y_{p}=\frac{\left(r_{2}^{2}-r_{3}^{2}-2x_{p}\left(x_{3}-x_{2}\right)-x_{2}^{2}+x_{3}^{2}+y_{3}^{2}\right)}{2y_{3}}.$$

After substituting Equation (4) into Equation (5), we obtain

(6) $$y_{p}=\frac{\left(r_{2}^{2}-r_{3}^{2}-\frac{r_{1}^{2}-r_{2}^{2}+x_{2}^{2}}{x_{2}}\left(x_{3}-x_{2}\right)-x_{2}^{2}+x_{3}^{2}+y_{3}^{2}\right)}{2y_{3}}.$$

From Equation (1), we express $z_{P}$ as

(7) $$z_{p}=\sqrt{r_{1}^{2}-x_{p}^{2}-y_{p}^{2}}.$$

Since points $T_{1}$, $T_{2}$, and $T_{3}$ are arranged in the reference plane in the form of an equilateral triangle with side length a, the following relationships can be substituted into Equations (4)–(6): $x_{3}=x_{2}/2$, $y_{3}={\sqrt{3}x}_{2}/2$*,* and $x_{2}=a$:

(8) $$x_{p}=\frac{r_{1}^{2}-r_{2}^{2}+a^{2}}{2a},$$

(9) $$y_{p}=\frac{r_{1}^{2}+r_{2}^{2}-2r_{3}^{2}+a^{2}}{2\sqrt{3}a},$$

(10) $$z_{p}=\sqrt{r_{1}^{2}-{\left(\frac{r_{1}^{2}-r_{2}^{2}+a^{2}}{2a}\right)}^{2}-{\left(\frac{r_{1}^{2}+r_{2}^{2}-2r_{3}^{2}+a^{2}}{2\sqrt{3}a}\right)}^{2}}.$$

When point P is displaced to point $P^{\prime }$, the distances $r_{1}$, $r_{2}$, and $r_{3}$ change accordingly. These changes in distance can be expressed in the following form:

(11) $$r_{1}^{\prime }=r_{1}+∆r_{1};\;\;\;\;r_{2}^{\prime }=r_{2}+∆r_{2};\;\;\;\;\;r_{3}^{\prime }=r_{3}+∆r_{3}\;,$$

where $∆r_{1}$, $∆r_{2}$, and $∆r_{3}$ represent the values measured by the inductive displacement transducers during the movement of point P to point $P^{\prime }$. The values of the individual displacement components of point P can be expressed as

$$u_{x}=x_{P}^{\prime }-x_{P},$$

(12) $$u_{x}=\frac{{\left(r_{1}+∆r_{1}\right)}^{2}-{\left(r_{2}+∆r_{2}\right)}^{2}+a^{2}}{2a}-\frac{r_{1}^{2}-r_{2}^{2}+a^{2}}{2a}\;,$$

$$u_{y}=y_{P}^{\prime }-y_{P},$$

(13) $$u_{y}=\frac{{\left(r_{1}+∆r_{1}\right)}^{2}+{\left(r_{2}+∆r_{2}\right)}^{2}-2{\left(r_{3}+∆r_{3}\right)}^{2}+a^{2}}{2\sqrt{3}a}-\frac{r_{1}^{2}+r_{2}^{2}-2r_{3}^{2}+a^{2}}{2\sqrt{3}a}\;,$$

$$u_{z}=z_{P}^{\prime }-z_{P},$$

(14) $$u_{z}={\left({\left(r_{1}+∆r_{1}\right)}^{2}-{\left(\frac{{\left(r_{1}+∆r_{1}\right)}^{2}-{\left(r_{2}+∆r_{2}\right)}^{2}+a^{2}}{2a}\right)}^{2}-{\left(\frac{{\left(r_{1}+∆r_{1}\right)}^{2}+{\left(r_{2}+∆r_{2}\right)}^{2}-2{\left(r_{3}+∆r_{3}\right)}^{2}+a^{2}}{2\sqrt{3}a}\right)}^{2}\right)}^{\frac{1}{2}}-{\left(r_{1}^{2}-{\left(\frac{r_{1}^{2}-r_{2}^{2}+a^{2}}{2a}\right)}^{2}-{\left(\frac{r_{1}^{2}+r_{2}^{2}-2r_{3}^{2}+a^{2}}{2\sqrt{3}a}\right)}^{2}\right)}^{\frac{1}{2}}.$$

Measurements are performed at two locations. The first location is situated beneath the ceiling on the northern side of reactor building and is marked with the capital letter S in Figure 4. The position of the measurement system and the orientation of the local coordinate system are shown in Figure 6.

The pipe displacement values at location S are obtained by substituting the parameters $r_{1}^{\left(S\right)}$, $r_{2}^{\left(S\right)}$, $r_{3}^{\left(S\right)}$, and a into Equations (12)–(14). Since the signal from the inductive displacement transducers is negative when the measured distance increases, the changes in distances $r_{1}^{\left(S\right)}$, $r_{2}^{\left(S\right)}$, and $r_{3}^{\left(S\right)}$ are introduced into Equations (12)–(14) as $\left(-S_{1}\right)$, $\left(-S_{2}\right)$, and $\left(-S_{3}\right)$, respectively.

In this way, the resulting expressions can be entered into the CATMAN Easy-AP software version 5.31 tool, which uses the measured three non-collinear displacements to calculate, in real time, the three displacement components of the selected point on the pipe within the coordinate system $\left(x_{S},{\;y}_{S},{\;z}_{S}\right)$:

(15) $$u_{x}^{\left(S\right)}=\frac{{\left(r_{1}^{\left(S\right)}-S_{1}\right)}^{2}-{\left(r_{2}^{\left(S\right)}-S_{2}\right)}^{2}+a^{2}}{2a}-\frac{{\left(r_{1}^{\left(S\right)}\right)}^{2}-{\left(r_{2}^{\left(S\right)}\right)}^{2}+a^{2}}{2a}\;,$$

(16) $$u_{y}^{\left(S\right)}=\frac{{\left(r_{1}^{\left(S\right)}-S_{1}\right)}^{2}+{\left(r_{2}^{\left(S\right)}-S_{2}\right)}^{2}-2{\left(r_{3}^{\left(S\right)}-S_{3}\right)}^{2}+a^{2}}{2\sqrt{3}a}-\frac{{\left(r_{1}^{\left(S\right)}\right)}^{2}+{\left(r_{2}^{\left(S\right)}\right)}^{2}-2{\left(r_{3}^{\left(S\right)}\right)}^{2}+a^{2}}{2\sqrt{3}a}\;,$$

(17) $$u_{z}^{\left(S\right)}={\left({\left(r_{1}^{\left(S\right)}-S_{1}\right)}^{2}-{\left(\frac{{\left(r_{1}^{\left(S\right)}-S_{1}\right)}^{2}-{\left(r_{2}^{\left(S\right)}-S_{2}\right)}^{2}+a^{2}}{2a}\right)}^{2}-{\left(\frac{{\left(r_{1}^{\left(S\right)}-S_{1}\right)}^{2}+{\left(r_{2}^{\left(S\right)}-S_{2}\right)}^{2}-2{\left(r_{3}^{\left(S\right)}-S_{3}\right)}^{2}+a^{2}}{2\sqrt{3}a}\right)}^{2}\right)}^{\frac{1}{2}}-{\left({\left(r_{1}^{\left(S\right)}\right)}^{2}-{\left(\frac{{\left(r_{1}^{\left(S\right)}\right)}^{2}-{\left(r_{2}^{\left(S\right)}\right)}^{2}+a^{2}}{2a}\right)}^{2}-{\left(\frac{{\left(r_{1}^{\left(S\right)}\right)}^{2}+{\left(r_{2}^{\left(S\right)}\right)}^{2}-2{\left(r_{3}^{\left(S\right)}\right)}^{2}+a^{2}}{2\sqrt{3}a}\;\right)}^{2}\right)}^{\frac{1}{2}}.$$

In the present case, the values measured during the installation of the system at position S are substituted into Equations (15)–(17):

$$r_{1}^{\left(S\right)}=775.00\;mm,\;\;\;\;r_{2}^{\left(S\right)}=775.00\;mm,\;\;\;\;r_{3}^{\left(S\right)}=705.00\;mm,\;\;\;\;a=770.70\;mm,$$

(18) $$u_{x}^{\left(S\right)}=\frac{{\left(775.00-S_{1}\right)}^{2}-{\left(775.00-S_{2}\right)}^{2}+770.70^{2}}{1541.40}-385.35,$$

(19) $$u_{y}^{\left(S\right)}=\frac{{\left(775.00-S_{1}\right)}^{2}+{\left(775.00-S_{2}\right)}^{2}-2\cdot {\left(705.00-S_{3}\right)}^{2}+770.70^{2}}{2669.78}-300.09,$$

(20) $$u_{z}^{\left(S\right)}={\left({\left(775.00-S_{1}\right)}^{2}-{\left(\frac{{\left(775.00-S_{1}\right)}^{2}-{\left(775.00-S_{2}\right)}^{2}+770.70^{2}}{1541.40}\right)}^{2}-{\left(\frac{{\left(775.00-S_{1}\right)}^{2}+{\left(775.00-S_{2}\right)}^{2}-2\cdot {\left(705.00-S_{3}\right)}^{2}+770.70^{2}}{2669.78}\right)}^{2}\right)}^{\frac{1}{2}}-601.73.$$

The second location is marked with the capital letter Q. The position of the measurement system and the orientation of the local coordinate system at this point are shown in Figure 4 and Figure 7.

The pipe displacement values at location Q are obtained by substituting the parameters $r_{1}^{\left(Q\right)}$, $r_{2}^{\left(Q\right)}$, $r_{3}^{\left(Q\right)}$, and a into Equations (12)–(14). The values $∆r_{1}$, $∆r_{2}$, and $∆r_{3}$ in Equations (12)–(14) are replaced by $\left(-Q_{1}\right)$, $\left(-Q_{2}\right)$, and $\left(-Q_{3}\right)$, respectively. The resulting expressions can then be entered into the CATMAN Easy-AP software tool for the real-time calculation of the three displacement components of the point Q within the coordinate system $\left(x_{Q},{\;y}_{Q},{\;z}_{Q}\right)$:

(21) $$u_{x}^{\left(Q\right)}=\frac{{\left(r_{1}^{\left(Q\right)}-Q_{1}\right)}^{2}-{\left(r_{2}^{\left(Q\right)}-Q_{2}\right)}^{2}+a^{2}}{2a}-\frac{{\left(r_{1}^{\left(Q\right)}\right)}^{2}-{\left(r_{2}^{\left(Q\right)}\right)}^{2}+a^{2}}{2a}\;,$$

(22) $$u_{y}^{\left(Q\right)}=\frac{{\left(r_{1}^{\left(Q\right)}-Q_{1}\right)}^{2}+{\left(r_{2}^{\left(Q\right)}-Q_{2}\right)}^{2}-2{\left(r_{3}^{\left(Q\right)}-Q_{3}\right)}^{2}+a^{2}}{2\sqrt{3}a}-\frac{{\left(r_{1}^{\left(Q\right)}\right)}^{2}+{\left(r_{2}^{\left(Q\right)}\right)}^{2}-2{\left(r_{3}^{\left(Q\right)}\right)}^{2}+a^{2}}{2\sqrt{3}a}\;,$$

(23) $$u_{z}^{\left(Q\right)}={\left({\left(r_{1}^{\left(Q\right)}-Q_{1}\right)}^{2}-{\left(\frac{{\left(r_{1}^{\left(Q\right)}-Q_{1}\right)}^{2}-{\left(r_{2}^{\left(Q\right)}-Q_{2}\right)}^{2}+a^{2}}{2a}\right)}^{2}-{\left(\frac{{\left(r_{1}^{\left(Q\right)}-Q_{1}\right)}^{2}+{\left(r_{2}^{\left(Q\right)}-Q_{2}\right)}^{2}-2{\left(r_{3}^{\left(Q\right)}-Q_{3}\right)}^{2}+a^{2}}{2\sqrt{3}a}\right)}^{2}\right)}^{\frac{1}{2}}-{\left({\left(r_{1}^{\left(Q\right)}\right)}^{2}-{\left(\frac{{\left(r_{1}^{\left(Q\right)}\right)}^{2}-{\left(r_{2}^{\left(Q\right)}\right)}^{2}+a^{2}}{2a}\right)}^{2}-{\left(\frac{{\left(r_{1}^{\left(Q\right)}\right)}^{2}+{\left(r_{2}^{\left(Q\right)}\right)}^{2}-2{\left(r_{3}^{\left(Q\right)}\right)}^{2}+a^{2}}{2\sqrt{3}a}\;\right)}^{2}\right)}^{\frac{1}{2}}.$$

In the present case, the values measured during the installation of the system at position Q are substituted into Equations (21)–(23):

$$r_{1}^{\left(Q\right)}=762.00\;mm,\;\;\;\;r_{2}^{\left(Q\right)}=778.00\;mm,\;\;\;\;r_{3}^{\left(Q\right)}=760.00\;mm,\;\;\;\;a=770.70\;mm,$$

(24) $$u_{x}^{\left(Q\right)}=\frac{{\left(762.00-Q_{1}\right)}^{2}-{\left(778.00-Q_{2}\right)}^{2}+770.70^{2}}{1541.40}-369.36,$$

(25) $$u_{y}^{\left(Q\right)}=\frac{{\left(762.00-Q_{1}\right)}^{2}+{\left(778.00-Q_{2}\right)}^{2}-2\cdot {\left(760.00-Q_{3}\right)}^{2}+770.70^{2}}{2669.78}-233.99,$$

(26) $$u_{z}^{\left(Q\right)}={\left({\left(762.00-Q_{1}\right)}^{2}-{\left(\frac{{\left(762.00-Q_{1}\right)}^{2}-{\left(778.00-Q_{2}\right)}^{2}+770.70^{2}}{1541.40}\right)}^{2}-{\left(\frac{{\left(762.00-Q_{1}\right)}^{2}+{\left(778.00-Q_{2}\right)}^{2}-2\cdot {\left(760.00-Q_{3}\right)}^{2}+770.70^{2}}{2669.78}\right)}^{2}\right)}^{\frac{1}{2}}-624.07.$$

All mathematical derivations were carried out by the authors.

### 2.1. Accuracy of the Measurement System

The accuracy of the designed measurement system was also examined. Since the inductive displacement transducers are mounted on both ends using miniature universal (cardan) joints, which exhibit a clearance between 0.1 mm and 0.2 mm, this factor alone introduces a measurement uncertainty in the order of approximately 0.5 mm.

**Figure 8:**
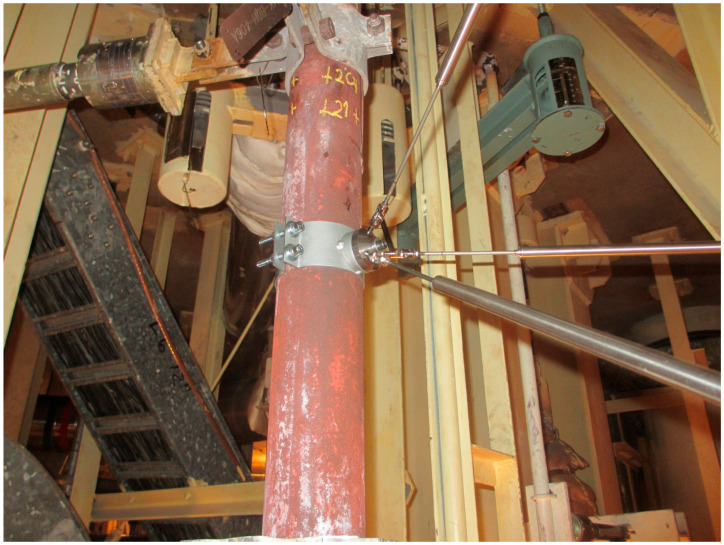
Mounting of the LVDT transducers on the pipe at the observation point S.

An additional source of measurement error arises from the geometry of the system. It is technically impossible to attach all three inductive transducers to exactly the same point on the pipe—where the observed point is defined—using universal joints for each sensor. As is shown in Figure 8, the attachment points of all three transducers on the pipe are therefore offset by 30 mm from the observed point. For small displacements, the geometric error of the system is practically negligible. However, as the displacement magnitude increases, this error becomes more significant. For large displacements—when the observed point reaches the extreme limits of the measurement range (±250 mm—Figure 3), the error can reach up to approximately 10 mm. Nevertheless, the relative measurement error remains below 5% throughout the measurements. A comprehensive analysis of the measurement error is presented in Appendix A.

At first glance, this might suggest that the overall accuracy of the measurement system is relatively low. However, it should be emphasized that the primary objective was not to develop a high-precision laboratory instrument but rather a robust measurement system with a large operational range (theoretically corresponding to a spherical volume with a radius of 250–300 mm). The system was designed to operate reliably under harsh environmental conditions while still providing dependable information about both the magnitude and direction of pipe displacements, with an accuracy fully sufficient for monitoring pipeline behavior under all operating regimes.

If higher accuracy was required, the same principle of measuring three non-collinear displacements could be retained while replacing the inductive displacement transducers with three Posiwire encoder-based sensors (manufactured by ASM). In such a configuration, the free ends of the three measuring wires could be attached to the same point on the pipe being monitored, thereby eliminating the geometric offset that is inherent in the current design. However, this alternative arrangement would introduce a new challenge: due to the size and specific geometry of the sensors, it would be extremely difficult to ensure the smooth winding and unwinding of the measuring wires in multiple displacement directions. The implementation of a fully rotatable mounting system for all three sensors would therefore be technically very complex.

An improved algorithm for calculating displacement components is currently under development, preserving the existing sensor configuration and nearly eliminating the error introduced by the offset installation of the LVDT sensors relative to the monitored point on the pipe.

### 2.2. Data Acquisition

As previously mentioned, the observation points S and Q, whose displacements are monitored by the described measuring system, are located inside the reactor building, which cannot be accessed during reactor operation (Figure 4).

This means that, inside the reactor building, there are inductive displacement transducers with their supporting structures and a measuring amplifier, whose functions include supplying power to the sensors, data acquisition, amplification, filtering, and conversion of analog signals into digital. These digital signals are then transmitted to a personal computer located outside the reactor building, in the plant’s processing computer room.

Due to the large distance between the measuring amplifier and the personal computer, communication between them takes place via an existing optical fiber cable, which is otherwise used for communication between the interior of the reactor building and the control room. Therefore, inside the reactor building, next to the measuring amplifier, an ethernet-to-optical signal converter is installed, while outside, in front of the personal computer, an optical-to-ethernet signal converter is installed.

**Figure 9:**
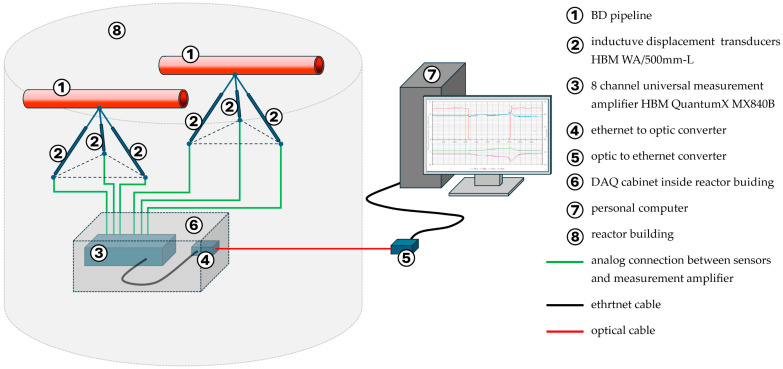
Measurement system setup.

**Figure 10:**
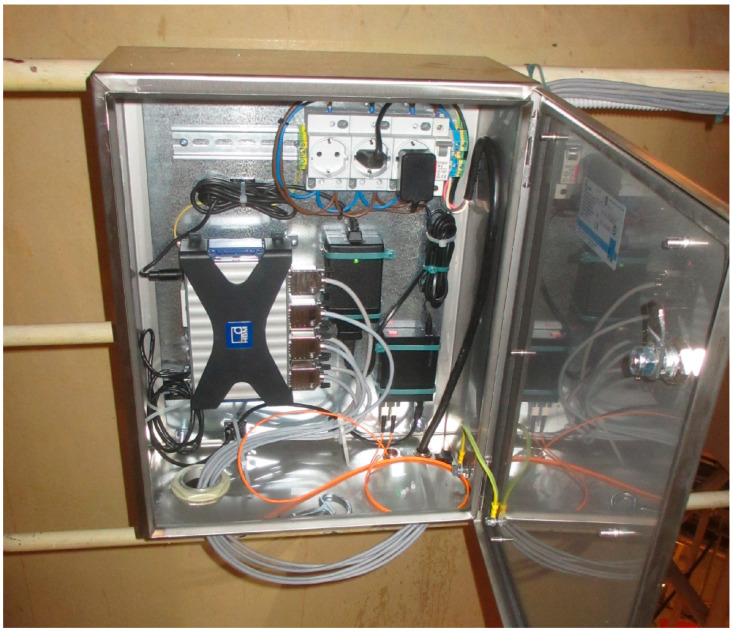
DAQ cabinet, containing DAQ unit, ethernet-to-optical converter, and power supplies.

Figure 9 shows the layout of the entire measurement system and Figure 10 shows a small cabinet, where the universal measurement amplifier QuantumX MX840B produced by Hottinger Baldwin Meßtechnik (HBM), Darmstadt, Germany, is located, together with the ethernet-to-optical converters and power supplies. CATMAN Easy-AP software, running on a personal computer, deals with data acquisition unit communication, recalculating measuring results and archiving all of the data in real time. The software application also allows more complex analysis (such as FFT, etc.), graphical representation, and data export. For monitoring unusual spurious displacements caused by system transients or seismic activity, plain time domain graphs are used.

### 2.3. Measurement Configuration

Before performing the measurements, it was necessary to prepare the measurement configuration using the CATMAN Easy-AP software tool. Since six inductive displacement transducers were connected to the measurement amplifier, the corresponding channels were labeled according to the independent variables appearing in Equations (18)–(20), as well as (24)–(26). The types of inductive sensors connected to each channel were also defined.

In addition, the computational channels were configured, the values of which are continuously calculated in real time based on the measured values from the input channels, following the mathematical relationships entered into the system (in this case, Equations (18)–(20), as well as (24)–(26)). For the automatic operation of the measurement system, it was necessary to define the measurement start mode (triggered or manual), as well as the method of storing both the measured and the calculated data (manual or automatic). Furthermore, the location on the computer’s storage medium, where the data would be saved, was specified, along with the procedure for automatically generating the file names for the recorded measurement data.

Considering the nature of the phenomena being monitored by the measurement system, a sampling frequency of 10 Hz was selected. The filenames were generated automatically, consisting of a short identifier followed by the date and time of acquisition. The files were saved to the computer’s hard drive every 10 min. The method and format of the real-time graphical display of both the measured and the computed results were also defined.

Engineers checked the system status several times per week and performed data backup and a basic event check once per week.

## 3. Results and Discussion

### 3.1. System Performance and Reliability

Three years after system installation, the reliability and robustness of the displacement monitoring system can now be meaningfully evaluated. Despite the fact that most of the measurement components (DAQ unit, sensors, and cabling) are located in a demanding operational environment characterized by elevated ambient temperatures and exposure to neutrons and radiation (n: 3 μSv/h, γ: 2 μSv/h, n + γ: 5 μS/h, corresponding to 0.22 SV/year), the system has operated continuously without functional interruptions. This confirms that the selection of the hardware components, measurement methodology, and installation approach was appropriate and effective.

The monitoring system is inspected every 18 months during scheduled plant outages to verify the sensor integrity, signal quality, and potential component degradation. Measurement data are continuously recorded and securely stored on a local hard drive. Under normal operating conditions, the collected displacement data are reviewed on a weekly basis, as well as following transient plant events such as operational tests, shutdowns, and startups.

### 3.2. Data Analysis

The displacement data can be further correlated with the relevant process parameters (e.g., flow rate, temperature, and pressure) obtained from the plant’s Process Information System (PIS). The integrated dataset enables a comprehensive analysis of pipeline behavior and facilitates the identification of potentially undesirable phenomena, such as localized boiling and water hammer events.

**Figure 11:**
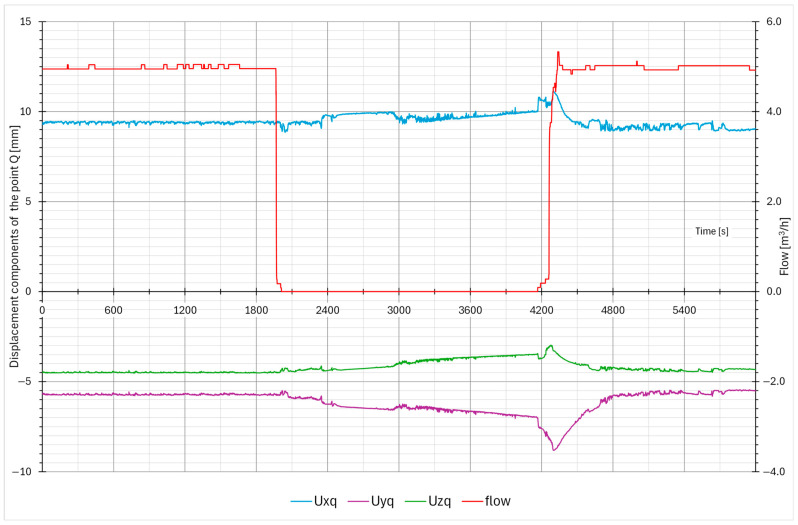
Typical pipe displacements during the BD system test (measuring point Q).

**Figure 12:**
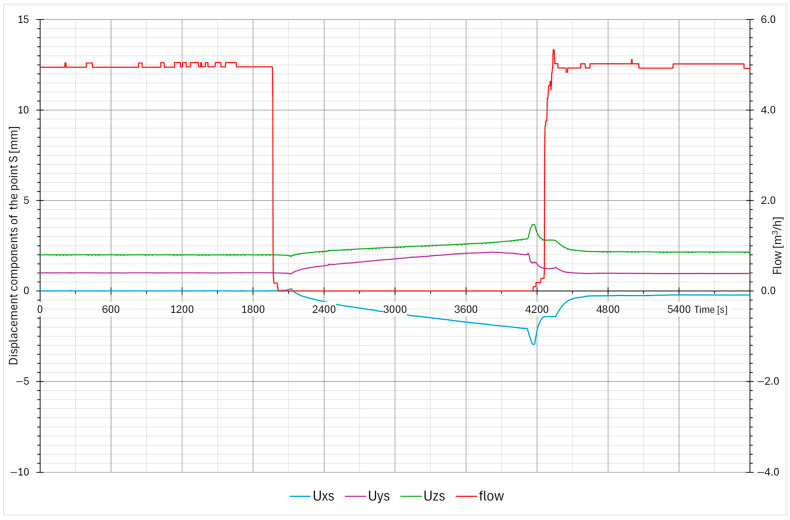
Typical pipe displacements during the BD system test (measuring point S).

**Figure 13:**
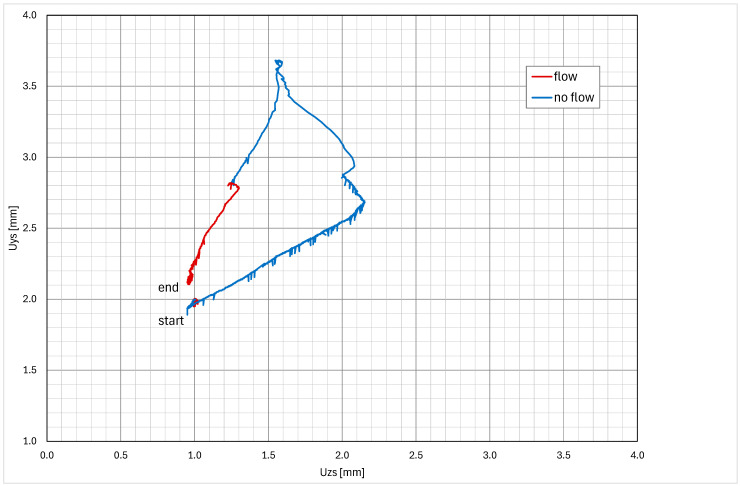
Typical Y–Z axis displacement during the BD system test (measuring point S).

**Figure 14:**
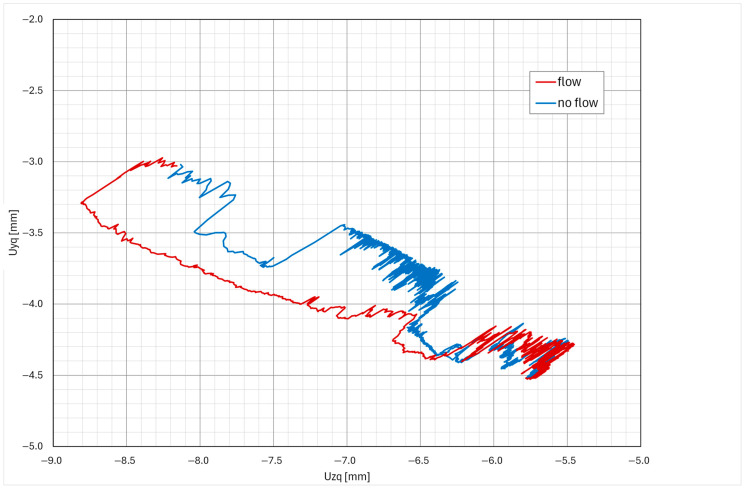
Typical Y–Z axis displacement during the BD system test (measuring point Q).

One of the basic data analyses is measuring the maximal displacement in each axis. The data are organized in weekly packets, and the maximum displacements are stored in a table. The “max–min” is the difference between the maximal and minimal pipe position in a one-week time window. The data are calculated for each axis and for both measuring points. In the case of any larger events, the data from the corresponding week are analyzed in detail. Table 1 presents the first few months of data from the startup of the measuring system. Excluding the plant startup phase—during which thermal expansion produced expected displacements associated with system heat-up—the largest measured displacements were recorded during week T18, coinciding with the performance of the blowdown system test (Figure 11, Figure 12, Figure 13 and Figure 14). Outside of this event, the BD system exhibited stable operating behavior, with displacement values remaining constant and within the expected limits.

The blowdown system test procedure involves the controlled closure of the flow control valves, resulting in gradual variations in both the flow rate and temperature within the piping [1]. These slow and continuous changes cause progressive cooling or heating of the pipe. When the valve reaches its fully closed position, a minor pressure transient occurs; however, its magnitude remains well below the threshold that could cause damage to the piping or its supporting structures.

The typical displacement responses recorded during this test are presented in Figure 11, Figure 12, Figure 13 and Figure 14. Figure 11 and Figure 12 show the displacement response in time domain. It is evident that when system flow is shut off, the pipe cools down and settles into a new position. After system flow restores, the pipe heats up and restores its approximate original position. Figure 13 and Figure 14 show the pipe displacement in the y–z plane (pipe cross-section). Graphs also represent system flow and no-flow conditions.

During the test, no abnormal transients (quick large movement of the pipe) were detected, confirming that the test procedure is appropriate and does not induce any fast and large pipe displacements. Any signs of those would imply that water hammering may be present, which causes significant dynamic stress on the pipes and support equipment, and should be avoided if possible. The plant test procedure is thus confirmed to be safe.

This study is limited to monitoring two points, but the system can be easily extended with additional DAQ units and sensors in case more measuring points are required. The system could also be upgraded with accelerometers, which measure acceleration in all three spatial directions. In theory, displacement can be obtained from accelerometer data by double integration [12]. This approach is suitable for detecting rapid movements (e.g., shocks), whereas slow thermal expansions cannot be reliably detected.

## 4. Conclusions

In high-energy piping systems commonly employed in power-generation facilities, including nuclear, thermal, and hydroelectric plants, the implementation of continuous displacement monitoring can provide valuable operational and diagnostic insight. Such systems support the verification of operating procedures, post-transient assessment, and the long-term observation of displacement trends relevant to structural integrity and fatigue-related considerations.

In this study, a computer-based, online, three-dimensional displacement monitoring system was developed and implemented at the Krško Nuclear Power Plant on the steam-generator blowdown system, a secondary auxiliary subsystem. Two monitoring points were installed on an 8-inch pipeline operating at an elevated temperature and pressure. The system is based on three non-collinear inductive displacement transducers mounted via universal joints, enabling full observability of spatial displacements while accommodating the geometric and access constraints of the reactor building.

The contribution of the presented work does not lie in the introduction of a new sensing principle, but in the experimental, system-level integration, long-term validation, and operational deployment of a three-dimensional displacement measurement concept under demanding nuclear power plant conditions. The revised manuscript now includes a quantitative uncertainty analysis and a pre-installation experimental validation against an independent reference, allowing the achievable measurement accuracy and its limitations to be transparently assessed.

The system has operated continuously for more than three years without major maintenance interventions, demonstrating the long-term operational stability and environmental durability of the hardware and measurement configuration. While geometric offsets at the attachment points introduce inherent kinematic complexity, their influence on measurement accuracy has been quantified and shown to remain within acceptable limits for the intended quasi-static monitoring application.

Overall, the presented system provides a robust and practical solution for continuous displacement monitoring in access-restricted and harsh environments, where conventional measurement approaches are difficult or impractical to deploy. The methodology and findings may be of interest for similar applications in nuclear and other energy-related facilities requiring long-term structural displacement monitoring.

## Figures and Tables

**Table 1:** Overview of maximum weekly displacement values. Dates are given in ISO 8601 format (YYYY–MM–DD).

Week	Start	End	Uxs [mm]	Uys [mm]	Uzs [mm]	Uxq [mm]	Uyq [mm]	Uzq [mm]	Comment
			Max−Min	Max−Min	Max−Min	Max−Min	Max−Min	Max−Min	
T1	2022–11–02	2022–11–07	15.1	8.8	10.5	36.8	33.9	19.6	plant heat-up
T2	2022–11–07	2022–11–14	1.3	1.0	0.8	3.8	2.8	1.5	
T3	2022–11–14	2022–11–21	0.2	0.2	0.2	0.5	0.6	0.3	
T4	2022–11–21	2022–11–28	0.2	0.5	0.5	0.4	0.3	0.2	
T5	2022–11–28	2022–12–05	0.3	1.0	0.4	0.3	0.3	0.3	
T6	2022–12–05	2022–12–12	1.7	2.1	1.7	2.7	2.7	1.3	
T7	2022–12–12	2022–12–19	2.1	1.9	2.4	3.1	4.6	1.8	
T8	2022–12–19	2022–12–26	0.3	0.6	0.2	0.4	0.4	0.2	
T9	2022–12–26	2023–01–02	0.4	0.9	0.4	1.0	0.6	0.3	
T10	2023–01–02	2023–01–09	0.2	0.4	0.2	0.6	0.4	0.3	
T11	2023–01–09	2023–01–16	0.1	0.2	0.1	0.1	0.2	0.1	
T12	2023–01–16	2023–01–23	0.2	0.4	0.3	0.4	0.6	0.2	
T13	2023–01–23	2023–01–30	0.2	0.4	0.2	0.4	0.3	0.2	
T14	2023–01–30	2023–02–06	0.4	0.7	0.4	0.6	0.4	0.3	
T15	2023–02–06	2023–02–13	0.1	0.2	0.2	0.2	0.2	0.1	
T16	2023–02–13	2023–02–20	0.3	0.5	0.2	0.4	0.4	0.2	
T17	2023–02–20	2023–02–27	0.4	0.8	0.5	1.0	0.7	0.3	
T18	2023–02–27	2023–03–06	7.0	3.6	6.2	11.2	16.5	7.0	BD test
T19	2023–03–06	2023–03–13	1.2	1.0	0.9	1.5	2.9	1.4	
T20	2023–03–13	2023–03–20	0.4	0.5	0.3	0.4	0.4	0.2	

**Figure A1:**
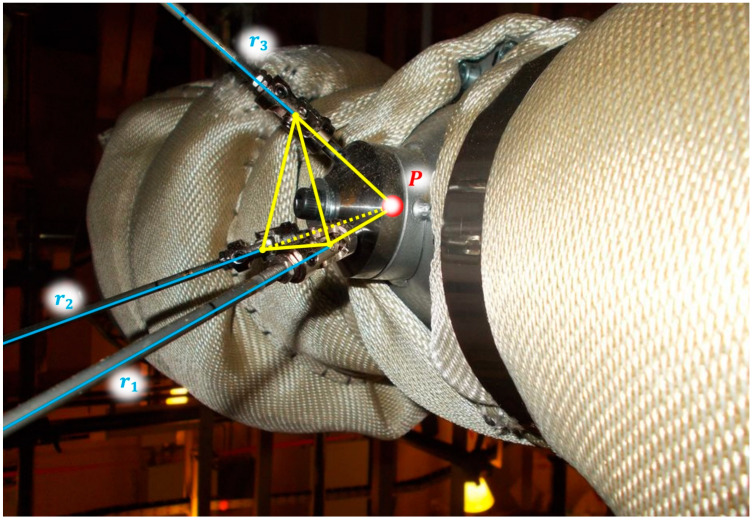
Offset installation of the inductive displacement transducers in the vicinity of the observed point, including a simplified geometric representation used in Figure A2 and Figure A3.

**Figure A2:**
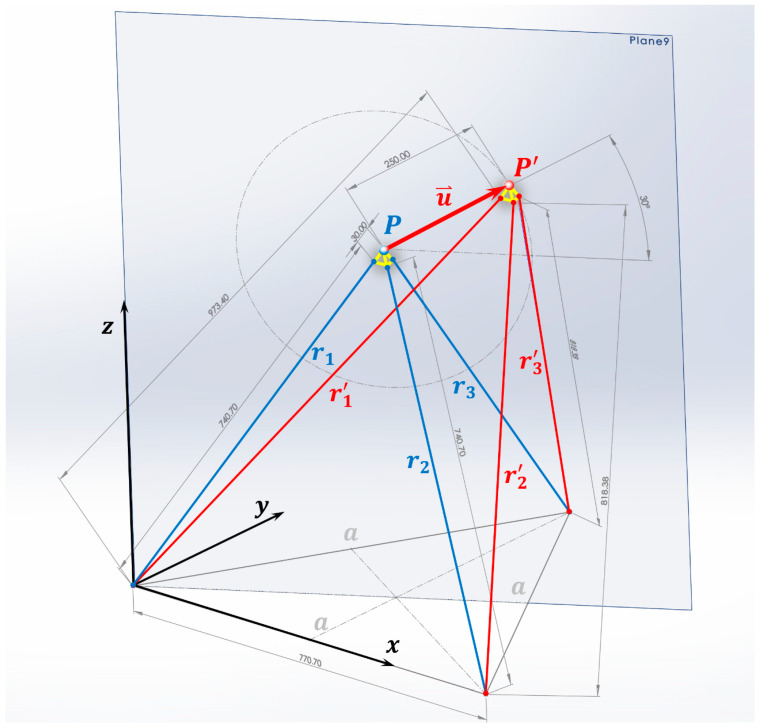
Three-dimensional sketch of the actual measurement system configuration in the initial and displaced position, including annotated dimensions for R=250 mm and Φ=30°.

**Figure A3:**
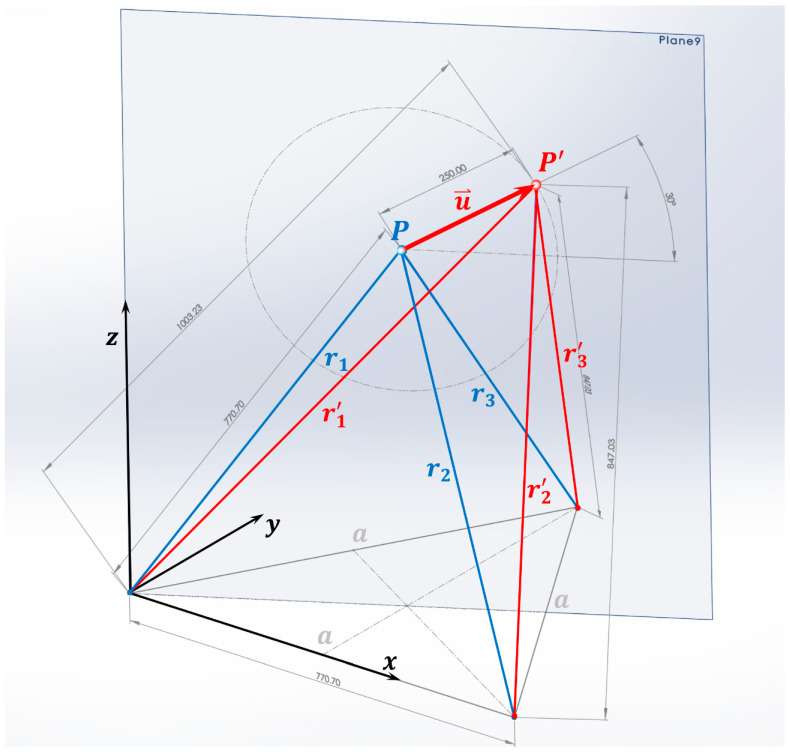
Three-dimensional sketch of the ideal measurement system configuration in the initial and displaced position, including annotated dimensions for R=250 mm and Φ=30°.

**Figure A4:**
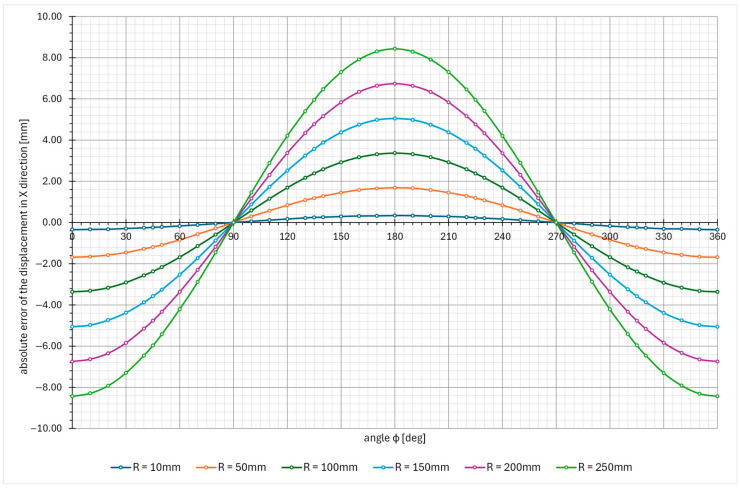
Absolute error of the X-direction displacement component of point P.

**Figure A5:**
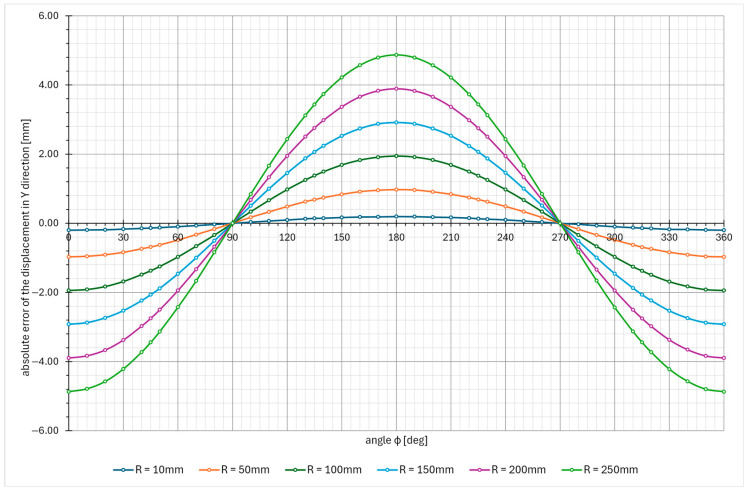
Absolute error of the Y-direction displacement component of point P.

**Figure A6:**
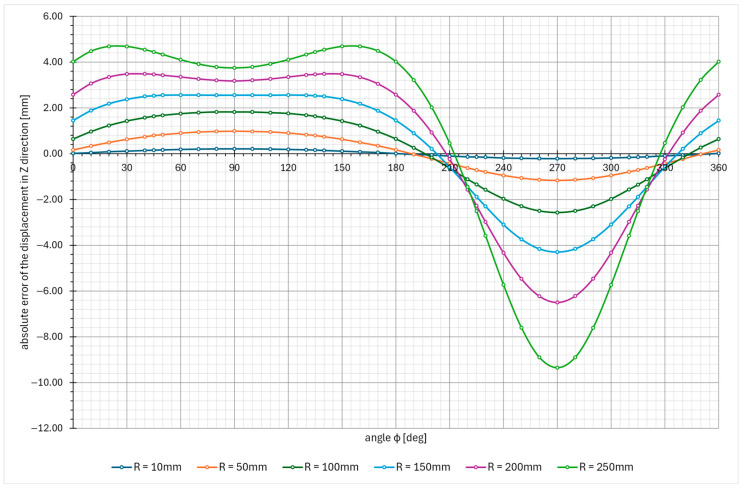
Absolute error of the Z-direction displacement component of point P.

**Figure A7:**
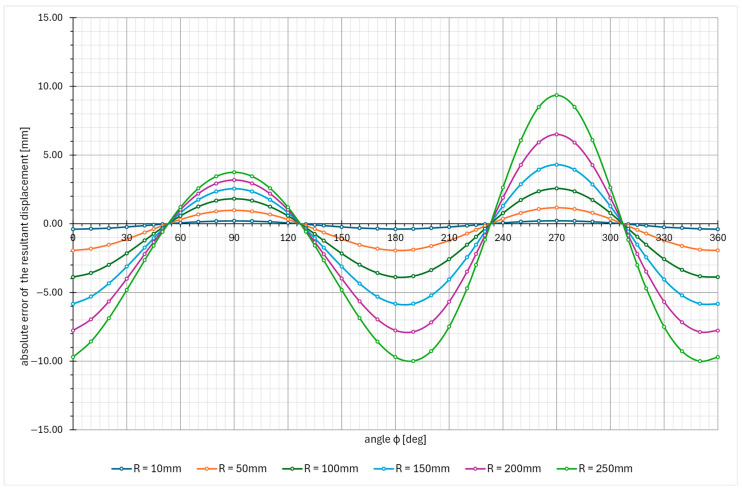
Absolute error of the resultant displacement of point P.

**Figure A8:**
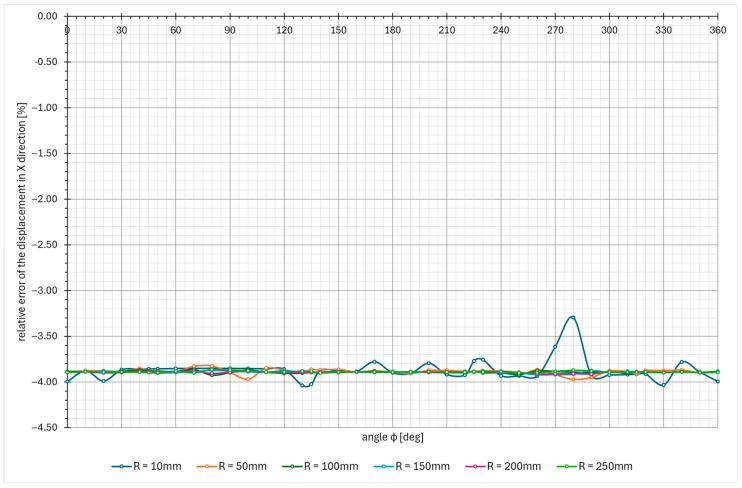
Relative error of the X-direction displacement component of point P.

**Figure A9:**
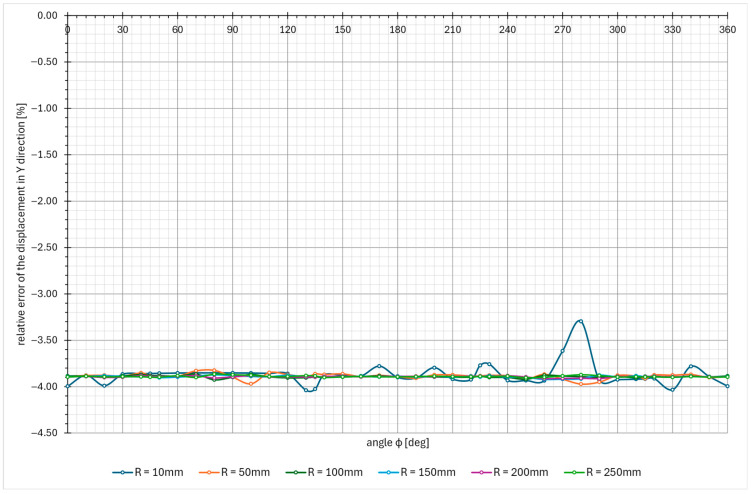
Relative error of the Y-direction displacement component of point P.

**Figure A10:**
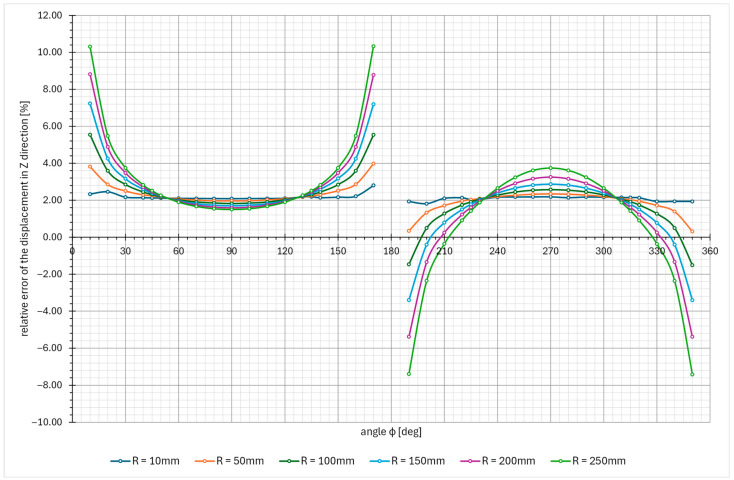
Relative error of the Z-direction displacement component of point P.

**Figure A11:**
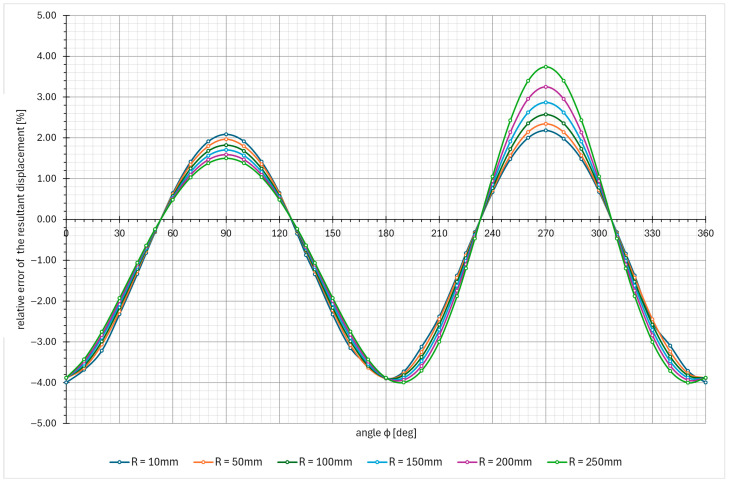
Relative error of the resultant displacement of point P.

## Data Availability

The data supporting the findings of this study are restricted by internal rules and regulations of the Krško Nuclear Power Plant and therefore cannot be made publicly available.

## Supplementary Materials

The following supporting information can be downloaded at: https://www.mdpi.com/article/10.3390/s26030895/s1, Table S1: Measurement error analysis, including numerical calculations and graphical results (Excel file).

## Abbreviations

The following abbreviations are used in this manuscript:

LVDT	Linear Variable Differential Transformer
HBM	Hottinger Baldwin Messtechnik
NPP	Nuclear Power Plant
DAQ	Data Acquisition
PIS	Process Information System
BD	Blowdown

## Appendix A. Measurement Error Analysis

The presented measurement configuration, based on three flexibly mounted inductive displacement transducers, enables the accurate and straightforward measurement of three-dimensional (3D) displacements of the observed point. Since the configuration does not include cantilever elements, measurement errors due to structural deformability are avoided. In addition, unlike the solutions presented in [8,9], the proposed system does not require the use of sensitive angle encoders.

A limitation of the configuration arises from the fact that three inductive displacement transducers cannot be independently and flexibly mounted at exactly the same point on the pipe circumference. Consequently, the attachment points of the transducers must be slightly offset from the observed point. In the implemented system, the transducers are mounted at the vertices of a fictitious tetrahedron with an edge length of 30 mm, as shown in Figure A1 and highlighted in yellow in the photograph of the transducer installation on the BD pipeline. The system is designed such that, in the initial configuration, the axes of all three inductive displacement transducers intersect at the observed point on the pipe. When the pipe is displaced from this initial position, the transducer axes are no longer aligned with the edges of the tetrahedron. As a result, an error is introduced in the calculated displacement components of the observed point. The absolute value of this error increases with increasing displacement magnitude and depends on the direction of motion.

The measurement error was quantified by comparing an ideal configuration, in which all three inductive displacement transducers are mounted at a single point on the pipe, with the actual configuration described above. The full measurement domain, shown in Figure 3 and corresponding to a sphere with a radius of 250 mm, was intersected by a plane, as illustrated in Figure A2 and Figure A3. The resulting intersection is a circular region within which the observed point P can move in the selected cross-sectional plane. The plane was chosen such that the values of $r_{2}^{\prime }$ and $r_{3}^{\prime }$ are equal, which simplifies the calculations without a loss of generality, since identical error behavior is obtained in all vertical planes passing through the initial position of point P.

The radius of the circle is denoted by R, and the counterclockwise angle between the horizontal line originating at the initial position of point P and the line connecting the initial and displaced positions (P and P′) is denoted by Φ. Using the formulations presented in Equations (12)–(14), the displacement components were calculated for both the actual and the ideal transducer configurations based on the values $r_{1}$, $r_{2}$, $r_{3}$, and $r_{1}^{\prime }$, $r_{2}^{\prime }$, $r_{3}^{\prime }$.

The analysis was performed for six circular paths with radii R=10,50,100,150,200, and 250 mm. Along each circle, the displaced point P′ occupied 40 positions defined by the angle Φ (Φ=0°, 10°, 20°, 30°, 40°, 45°, 50°, 60°, 70°, …). The calculations are provided in the Supplementary File Measurement_error.xlsx. For each position, the input parameters were $r_{1}^{\prime }$ and $r_{2}^{\prime }$, with $r_{3}^{\prime }$ = $r_{2}^{\prime }$. These values were obtained from 3D sketches created in SolidWorks version 2023 for each position of point P. For every combination of R and Φ, two sketches were generated: one representing the actual configuration and one representing the ideal configuration. The corresponding values of $r_{1}^{\prime }$ and $r_{2}^{\prime }$, were extracted directly from the dimensioned sketches. An example of R=250 mm and Φ=30° is shown in Figure A2 and Figure A3.

For each configuration, the displacement components $u_{x}$, $u_{y}$, and $u_{z}$ as well as the resultant displacement u, were calculated. The measurement error was then obtained by comparing the displacement components calculated for the actual and ideal configurations. This error represents the effect of offsetting the transducer attachment points from the observed point in order to achieve a technically feasible installation.

The results are presented graphically in Figure A4, Figure A5, Figure A6, Figure A7, Figure A8, Figure A9, Figure A10 and Figure A11. The absolute measurement error increases with increasing displacement from the initial position P. For the largest considered radius (R=250 mm), the absolute error in the X-direction displacement component ranges from −8.2 mm to 8.2 mm (Figure A4), in the Y-direction from −4.8 mm to 4.8 mm (Figure A5), and in the Z-direction from −8.6 mm to 4.8 mm (Figure A6). The absolute error of the resultant displacement u remains within ±10 mm (Figure A7). In contrast, for the smallest radius (R=10 mm), the absolute errors are significantly smaller: −0.35 mm to 0.34 mm for $u_{x}$, −0.20 mm to 0.20 mm for $u_{y}$, −0.22 mm to 0.22 mm for $u_{z}$, and −0.40 mm to 0.22 mm for the resultant displacement u.

The relative errors of the displacement components and the resultant displacement show only a weak dependence on displacement magnitude. The relative errors in the X and Y components remain close to −4% (Figure A8 and Figure A9), the relative error in the Z component varies between 1.5% and 4.0% (Figure A10), and the relative error of the resultant displacement u lies within ±4.0% (Figure A11).

Except in the vicinity of singular points in the relative error distribution of the Z-direction displacement component, the relative measurement error remains below 5%, which satisfies the requirement specified in the project task.

Prior to installation in the reactor building, the measurement concept and achievable accuracy were experimentally verified under controlled conditions. A robotic total station (Leica TS50, angular accuracy 0.5″) was used as an independent reference system. A geodetic prism was positioned at the intended attachment location of the monitoring system, and its three-dimensional trajectory was recorded while controlled displacements were imposed. The same displacement paths were simultaneously measured using the inductive transducer configuration.

The comparison showed that the reconstructed displacements agreed with the reference measurements within ±5%, which was defined as the acceptance criterion for deployment. This validation confirmed that the proposed measurement concept provides reliable displacement estimates within the accuracy required for the intended long-term monitoring application.
